# Interactions between chronic kidney disease and hyperuricemia in children: mechanisms, clinical manifestations and management updates

**DOI:** 10.3389/fped.2026.1975951

**Published:** 2026-10-01

**Authors:** Huang Xiaoxia, Liu Yuhua

**Affiliations:** Guangzhou First People’s Hospital, South China University of Technology, Guangzhou, Guangdong, China

**Keywords:** children, chronic kidney disease, clinical management, hyperuricemia, renal injury, uric acid

## Abstract

A bidirectional pathogenic interplay exists between pediatric chronic kidney disease (CKD) and hyperuricemia (HUA). Hyperuricemia serves as an independent risk factor for the onset and progression of CKD, while also representing a common complication arising from impaired renal excretory function. Amid the rising global prevalence of pediatric CKD and growing evidence implicating metabolic disturbances in renal injury, the reciprocal pathological mechanisms underlying uric acid–mediated kidney damage have emerged as a critical research focus. Elevated serum uric acid induces renal tubulointerstitial injury through oxidative stress, systemic inflammatory cascades, and endothelial dysfunction. Conversely, diminished uric acid excretion resulting from CKD further aggravates hyperuricemia, establishing a self-perpetuating vicious cycle. However, epidemiological evidence specific to the pediatric population remains limited, and current clinical management strategies are largely extrapolated from adult studies, lacking evidence-based guidelines tailored to the unique growth and developmental characteristics of children. This review systematically summarizes the epidemiological features, molecular pathogenic mechanisms, clinical implications, and therapeutic approaches for HUA comorbid with pediatric CKD. We specifically highlight uric acid–driven renal injury pathways, the accelerating effect of HUA on CKD progression, and age-specific challenges in pediatric diagnosis and standardized intervention. By integrating recent advances in preclinical and clinical research, this review aims to provide a theoretical foundation for the early screening, timely diagnosis, and individualized standardized management of hyperuricemia in children with CKD.

## 1 Introduction

The global incidence of pediatric chronic kidney disease (CKD) has risen steadily, emerging as a critical public health threat to children’s long-term health (1, 2). Metabolic disorders constitute a core pathological driver throughout CKD progression, among which hyperuricemia (HUA) has attracted growing clinical attention as a frequent comorbidity. Cumulative evidence indicates that elevated serum uric acid is not merely a secondary biomarker of declining renal function, but directly facilitates glomerulosclerosis and tubulointerstitial fibrosis via multiple pathological pathways. Clinical investigations in adults have validated HUA as an independent risk factor for CKD onset and deterioration. A Chinese multicenter cohort of hospitalized adult CKD patients further confirmed that hyperuricemia predicts early renal injury independently (3). Collectively, these findings substantiate the tight bidirectional correlation between uric acid metabolism and chronic kidney damage.

Recent mechanistic studies have deciphered the multifaceted pathogenic pathways of uric acid in CKD progression. High serum urate activates inflammatory signaling, provokes oxidative stress and impairs vascular endothelial integrity, ultimately driving tubulointerstitial fibrosis and glomerular sclerosis. Both animal models and human clinical specimens demonstrate that uric acid stimulates vascular smooth muscle proliferation and overactivates the renin–angiotensin system (RAS), aggravating progressive renal parenchymal damage (4, 5). Distinct from adults, the etiology and clinical phenotypes of HUA in pediatric CKD exhibit prominent age-specific characteristics. Beyond dietary factors and iatrogenic drug exposure, inherited tubular disorders represent a dominant pediatric-specific trigger of hyperuricemia. Familial juvenile hyperuricemic nephropathy (FJHN), an autosomal dominant disorder caused by UMOD gene mutations, is characterized by early-onset hyperuricemia, reduced fractional urate excretion and irreversible progressive renal failure (6, 7). Other inborn metabolic diseases including methylmalonic acidemia (MMA) also predispose children to juvenile hyperuricemia and clinical gout (8). Hereditary etiologies carry far greater weight in pediatric HUA compared with adults, rendering the pediatric causal spectrum more heterogeneous and complex.

Given the intricate reciprocal relationship between pediatric CKD and HUA, as well as unique pediatric pathogenic and clinical features, a comprehensive review of this comorbidity is clinically imperative. This paper systematically dissects the interplay between pediatric CKD and HUA from four dimensions: epidemiological characteristics and causative factors, molecular pathogenic mechanisms, clinical diagnostic evaluation, and individualized therapeutic regimens. In epidemiological sections, we summarize HUA prevalence across CKD stages and longitudinal associations with renal function decline. Mechanistically, we elaborate on inflammatory, oxidative and vascular injury cascades through which uric acid induces renal lesions. For clinical evaluation, we highlight differential diagnostic workflows for pediatric HUA, with particular emphasis on screening inherited tubular nephropathies. In therapeutic sections, we synthesize current evidence and safety caveats of urate-lowering agents in pediatric CKD populations. By synthesizing up-to-date translational and clinical research, this review establishes standardized clinical management workflows to slow CKD progression and improve long-term outcomes of affected children.

## 2 Epidemiological features and etiology of hyperuricemia in pediatric CKD

### 2.1 Prevalence and modifiable risk factors

The prevalence of hyperuricemia among children with CKD ranges from 20% to 50%, rising markedly alongside advancing CKD stages, with the highest burden observed in stage 4–5 patients (9, 10). This gradient is primarily attributed to progressive nephron loss and impaired renal urate clearance. Multiple independent risk factors predispose children to HUA, including male sex, obesity, high-purine dietary patterns and chronic diuretic administration. Inherited tubular disorders such as familial juvenile hyperuricemic nephropathy (FJHN) also constitute non-negligible primary causes. Notably, preterm and low-birth-weight infants exhibit incomplete nephrogenesis, conferring substantially elevated HUA risk that accelerates subsequent CKD deterioration. A national registry-based cohort of Iranian pediatric CKD patients identified congenital anomalies of the kidney and urinary tract (CAKUT) as the leading etiology (40.01%), followed by glomerular diseases (19.00%) (11). Analysis of nationwide pediatric hospitalization data in China demonstrated secondary nephropathies as the most prevalent CKD trigger (37.95%), with CAKUT ranking second (24.61%) (12). These population-based epidemiological data underscore the necessity of systematic screening and targeted intervention for HUA risk factors to retard pediatric CKD progression.

### 2.2 Etiological classification and genetic background

Pediatric hyperuricemia is categorized into primary and secondary subtypes according to pathogenic origins. Primary HUA predominantly arises from germline mutations in renal urate transporters: pathogenic variants in URAT1 (SLC22A12) or GLUT9 (SLC2A9) lead to reduced tubular urate excretion and hypoexcretory hyperuricemia. Secondary HUA develops secondary to CKD, tumor lysis syndrome, or adverse effects of specific medications (13). Genetically mediated FJHN driven by UMOD mutations represents a major inherited cause of tubulointerstitial nephropathy. Such hereditary hyperuricemia invariably accompanies progressive tubulointerstitial fibrosis; early genetic screening enables timely intervention to curb irreversible renal decline. The advent of high-throughput sequencing has revolutionized the diagnostic landscape of pediatric CKD, implying that the true incidence of childhood nephropathies may be substantially higher than current clinical estimates (14). A single-center cohort from Syria reported CAKUT as the dominant CKD etiology (79%), inherited nephropathies accounting for 12%, and glomerular diseases for 9% (15). Precise classification of HUA etiologies and comprehensive characterization of genetic susceptibility facilitate precision diagnosis and personalized therapeutic strategies for affected children.

### 2.3 Longitudinal correlation between HUA and pediatric CKD progression

Cross-sectional and long-term cohort studies consistently confirm a positive linear correlation between serum uric acid levels and the rate of renal function deterioration in pediatric CKD patients (10, 16). Children with hyperuricemia exhibit a 1.5–3 fold higher risk of progressing to end-stage kidney disease (ESKD) compared with normouricemic counterparts. Direct tubular cytotoxicity induced by excess urate has been pathologically validated in pediatric renal specimens, manifesting as proximal tubular epithelial apoptosis and mitochondrial dysfunction. A predictive analysis of CKD risk among obese adolescents demonstrated that elevated serum uric acid independently correlates with incident CKD, regardless of baseline estimated glomerular filtration rate (eGFR) (17). Additionally, pediatric CKD confers markedly elevated cardiovascular risk, wherein hyperuricemia functions as an independent cardiovascular risk amplifier (18). Longitudinal follow-up further revealed that progressive CKD impairs neurocognitive development: a Korean multicenter cohort reported a mean full-scale intelligence quotient (IQ) of 91 ± 19 among CKD children, with 24.7% presenting IQ <80, and advanced CKD stages correlated significantly with poorer cognitive performance (19). Collectively, these findings demonstrate that HUA is not merely a passive biomarker of CKD severity, but an independent modifiable driver of renal function loss, highlighting the clinical value of early urate-lowering intervention.

## 3 Molecular mechanisms and clinical consequences of HUA in pediatric CKD

### 3.1 Uric acid-mediated renal injury signaling cascades

Uric acid triggers renal parenchymal injury via synergistic activation of multiple interwoven signaling pathways. First, urate overactivates the intrarenal renin–angiotensin system (RAS), upregulating angiotensin II expression to induce intraglomerular hypertension and mesangial cell proliferation, initiating irreversible renal damage (20). Second, uric acid drives oxidative stress and activates NF-*κ*B inflammatory signaling, promoting robust secretion of pro-inflammatory cytokines including TNF-α and IL-6. These mediators trigger tubular epithelial–mesenchymal transition (EMT), accelerating irreversible tubulointerstitial fibrosis (21). When serum urate exceeds solubility thresholds, insoluble monosodium urate crystals form within renal tissue; such crystals act as danger-associated molecular patterns (DAMPs) recognized by the NLRP3 inflammasome, subsequently activating caspase-1 to mediate pyroptosis and macrophage infiltration, establishing a self-reinforcing cycle of crystal deposition, inflammation and renal fibrosis (22). Notably, preclinical animal studies clarified that isolated asymptomatic hyperuricemia without intrarenal crystalluria fails to drive CKD progression; only intrarenal urate crystal deposition initiates obstructive tubular lesions, interstitial inflammatory infiltration and progressive fibrosis through the above inflammatory axes (23).

### 3.2 Dual detrimental effects of HUA on renal and cardiovascular systems

In children with CKD, hyperuricemia imposes dual adverse impacts: accelerating renal function decline and exacerbating cardiovascular end-organ damage. Population data indicate that each 1 mg/dL elevation in serum uric acid increases CKD progression risk by approximately 20%, with this association independent of pediatric age and sex (24). Uric acid inhibits endothelial nitric oxide (NO) synthesis, disrupting vasomotor homeostasis and generating a prothrombotic state, thereby elevating risks of pediatric hypertension and left ventricular hypertrophy (25). Ambulatory blood pressure monitoring (ABPM) abnormalities, a hallmark of early cardiovascular damage, are independently associated with hyperuricemia in pediatric CKD cohorts (26). Concurrent diabetes mellitus amplifies the adverse effects of HUA, synergistically elevating risks of ESKD and all-cause mortality (27). Among children with sickle cell disease (SCD), hyperuricemia correlates significantly with reduced eGFR, verifying its nephrotoxic effect across heterogeneous pediatric nephropathy etiologies (24).

### 3.3 Pediatric-specific clinical manifestations and diagnostic pitfalls

Clinical presentations of pediatric HUA differ substantially from adult counterparts, frequently leading to delayed or misdiagnosis. Most affected children remain asymptomatic; merely 15% develop clinical gout or uric acid nephrolithiasis, with crystal formation risk markedly elevated under dehydration or alkaline urine conditions (28). In early-stage renal insufficiency, pediatric HUA is predominantly characterized by reduced fractional excretion of urate (FeUA), a phenotype distinct from adults—adult urate retention mainly stems from reduced glomerular filtration, whereas pediatric HUA primarily arises from impaired tubular urate secretion (24). This mechanistic discrepancy prohibits direct extrapolation of adult diagnostic criteria to children. Moreover, serum uric acid reference ranges vary drastically with chronological age, converging toward adult thresholds only after puberty; neglect of developmental physiological variation causes frequent missed or overdiagnosis (29). Inherited disorders including HPRT1-mutated Lesch-Nyhan syndrome and UMOD-related autosomal dominant tubulointerstitial kidney disease (ADTKD) frequently present with isolated juvenile hyperuricemia and gout, often misdiagnosed in the absence of typical neurological manifestations (30, 31).

## 4 Management strategies and research prospects for HUA in pediatric CKD

### 4.1 Lifestyle and nutritional intervention

Lifestyle modification constitutes the foundational intervention for HUA in pediatric CKD, aiming to lower serum urate and retard renal deterioration via dietary adjustment and regular physical activity. Restricted purine intake serves as the core nutritional principle: red meat, seafood and sugar-sweetened beverages should be limited, while low-fat dairy products and whole grains are recommended. Dietary polysaccharides, flavonoids and polyphenols exert urate-lowering effects through xanthine oxidase inhibition, modulation of renal urate transporters and remodeling of intestinal microbiota (32). Adequate daily fluid intake (1.5–2 L, adjusted for body weight and renal status) facilitates urate excretion and prevents uric acid stone formation; dehydration strongly correlates with acute hyperuricemia—among children with acute gastroenteritis, moderate-severe dehydration yields a 95.0% HUA prevalence versus merely 10.9% in mild dehydration cohorts (33). Weight reduction via structured physical activity is prioritized for overweight and obese children, as adipose tissue augments endogenous urate biosynthesis. Population data from US adolescents confirm robust correlation between obesity and hyperuricemia across ethnic and gender subgroups (34). Additionally, plant-based vegan diets correlate with reduced CKD risk among hyperuricemic patients, highlighting renoprotective potential of low-purine vegetarian dietary patterns (35).

### 4.2 Pharmacotherapeutic principles and regimens

Given the limited pediatric evidence, current guidelines adopt a conservative stance toward urate-lowering therapy (ULT) in asymptomatic hyperuricemia. In children without gout, urolithiasis, or established renal injury directly attributable to hyperuricemia, routine initiation of ULT is generally not recommended, largely because the renal benefits of lowering serum uric acid remain uncertain and are outweighed by potential drug toxicity. The most concerning risk is the HLA-B*5801-associated severe cutaneous adverse reaction to allopurinol, which is markedly more prevalent in Asian populations and warrants screening or avoidance in high-risk children. Consequently, treatment decisions should be individualized, prioritizing lifestyle modification and management of modifiable metabolic factors before considering pharmacologic intervention.

Pharmacological urate-lowering therapy is initiated when lifestyle intervention fails to achieve target serum uric acid levels. Allopurinol remains the first-line agent for pediatric HUA, suppressing *de novo* urate synthesis via xanthine oxidase inhibition. A retrospective analysis of 1753 hyperuricemic children demonstrated that standard initial allopurinol doses (50–100 mg daily) normalize serum urate in most patients, with dose escalation to 200–300 mg daily required for severely obese adolescents (36). Dose reduction is mandatory for CKD children with eGFR <30 mL/min/1.73m². Febuxostat possesses potent urate-lowering efficacy, yet pediatric clinical experience remains limited. A Japanese phase II multicenter trial of febuxostat in children with hyperuricemia/gout reported target urate control (≤6.0 mg/dL) in 63.3% of participants after 26 weeks of treatment, with no mandatory dose adjustment for mild-to-moderate renal impairment (37). Nevertheless, insufficient long-term pediatric safety data restrict febuxostat to second-line use for children intolerant to allopurinol (38). Uricosuric agents such as probenecid lose efficacy when eGFR falls below 30 mL/min and carry limited pediatric clinical data, requiring cautious administration. Citrate salts are indicated for patients with uric acid nephrolithiasis or urinary pH <6.0 to alkalinize urine, targeting urinary pH 6.5–7.0, with close monitoring to avoid metabolic alkalosis.

### 4.3 Surveillance and long-term management core principles

Long-term integrated management of pediatric CKD-HUA requires regular multimodal monitoring and holistic risk evaluation. Serum uric acid, renal function, fractional urate excretion and ambulatory blood pressure should be tested every 3–6 months to assess therapeutic response and CKD progression. Estimated glomerular filtration rate correlates positively with urinary purine and pyrimidine excretion, implying that renal function status must be incorporated to avoid misinterpretation of urate excretion indices (39). Clinicians must maintain high vigilance for severe allopurinol hypersensitivity reactions, particularly Stevens-Johnson syndrome, which exhibits markedly higher incidence among carriers of HLA-B*5801; pre-treatment genetic screening is recommended for high-risk populations. Hyperuricemia independently drives hypertension via oxidative stress and enhanced xanthine oxidoreductase activity; accordingly, comprehensive cardiovascular risk mitigation is mandatory for affected children, including strict blood pressure and lipid control and avoidance of tobacco exposure (40). Real-world cross-sectional data from Japan indicate rising urate-lowering therapy utilization with advancing pediatric age, with agent selection individualized based on age, sex and concomitant complications (41).

### 4.4 Emerging therapeutic directions

Advancements in understanding HUA pathophysiology have uncovered multiple promising novel therapeutic targets. Urate transporter inhibitors such as lesinurad demonstrate robust urate-lowering capacity in adult cohorts, yet dedicated pediatric clinical trials remain uninitiated. Recombinant urate oxidase is primarily approved for tumor lysis syndrome (TLS), producing rapid profound serum urate reduction. Retrospective pediatric acute kidney injury studies confirmed that rasburicase normalizes serum urate within 24 h and partially recovers eGFR (42). Nevertheless, rasburicase carries critical safety risks for pediatric CKD patients: severe hypersensitivity reactions and life-threatening methemoglobinemia/hemolytic anemia in glucose-6-phosphate dehydrogenase (G6PD)-deficient children (43). Targeted inhibition of NLRP3 inflammasome and oxidative stress pathways represents another promising therapeutic frontier. Single-cell transcriptomic profiling of pediatric gout identified CD14 + monocytes and DN T cells as core inflammatory mediators, highlighting the TNF-α/NF-*κ*B axis as druggable targets (44). Additionally, intestinal microbiota modulates systemic urate homeostasis: specific Lactobacillus strains degrade dietary purines and circulating uric acid, laying the groundwork for probiotic-based adjuvant therapy, particularly for advanced CKD patients with limited conventional pharmacological options (45). These emerging modalities provide innovative avenues for future therapeutic development in pediatric HUA complicated with CKD.

## 5 Conclusion

The bidirectional pathogenic interaction between pediatric CKD and hyperuricemia has been universally validated by translational and clinical research, representing a core pathological node and major clinical challenge in nephrology practice. Mechanistically, uric acid induces progressive renal tubular and glomerular damage via coordinated activation of inflammatory cascades, oxidative stress and intrarenal RAS overactivation, while simultaneously exacerbating systemic cardiovascular burden to form a self-perpetuating vicious cycle. Despite minor discrepancies across studies regarding urate toxic thresholds and relative contribution of individual injury pathways, the integrated evidence chain is robust to support rational clinical intervention. Current clinical consensus prioritizes universal early screening and risk-stratified targeted intervention. Regular serum uric acid surveillance should be integrated into routine CKD follow-up for all children, especially those with renal dysfunction, obesity or metabolic syndrome. Lifestyle modification including low-purine diets, sustained adequate hydration and weight control forms the irreplaceable first-line intervention, yet therapeutic efficacy is heavily constrained by pediatric treatment adherence. Allopurinol is endorsed as first-line pharmacotherapy with robust evidence for urate reduction and renal protection, though mandatory dose adjustment for impaired eGFR and pre-screening for hypersensitivity risk are required (7). Controversies persist regarding routine urate-lowering therapy for asymptomatic hyperuricemic children, as long-term pediatric safety and sustained renoprotective efficacy remain incompletely characterized.

From a translational perspective, this field is characterized by well-elucidated core mechanisms, fragmented pediatric clinical evidence and insufficient clinical translation. Heterogeneity in study design, sample size, follow-up duration and confounding variable adjustment generates divergent conclusions on whether HUA acts as an innocent bystander or causal driver of CKD progression. A balanced clinical perspective acknowledges dual roles of HUA: a sensitive early warning biomarker and a modifiable independent accelerator of renal deterioration. Clinical decision-making must rely on individualized risk stratification rather than rigid extrapolation of adult diagnostic and therapeutic thresholds.

Future research priorities should fill critical gaps in pediatric-specific clinical evidence, including pharmacokinetics, long-term safety and renoprotective efficacy of novel urate-lowering agents in children. Large-scale multicenter prospective randomized controlled trials are urgently required to upgrade the quality of pediatric clinical evidence. Further investigations are warranted to develop early renal injury predictive models incorporating urinary biomarkers and validate adjuvant non-pharmacological interventions such as microbiota-targeted probiotics. Ultimately, standardized management of pediatric CKD-HUA should shift from isolated urate reduction to comprehensive multi-target renal protection, integrating metabolic, inflammatory and cardiovascular risk mitigation to optimize long-term pediatric renal and cardiovascular outcomes.
